# Comparative analysis of floc characteristics and microbial communities in anoxic and aerobic suspended growth processes

**DOI:** 10.1002/wer.10822

**Published:** 2022-12-21

**Authors:** Huanqi He, Avery L. Carlson, Per Halkjær Nielsen, Jizhong Zhou, Glen T. Daigger

**Affiliations:** ^1^ Department of Civil and Environmental Engineering University of Michigan Ann Arbor Michigan USA; ^2^ Center for Microbial Communities, Department of Chemistry and Bioscience Aalborg University Aalborg Denmark; ^3^ Institute for Environmental Genomics, Department of Microbiology and Plant Biology, School of Civil Engineering and Environmental Sciences, and School of Computer Science University of Oklahoma Norman Oklahoma USA

**Keywords:** anoxic, community assembly, flocs, microbial diversity, suspended growth, temporal dynamics

## Abstract

A fully anoxic suspended growth process is an appealing alternative to conventional activated sludge (AS) due to considerable aeration reduction and improved carbon processing efficiency for biological nutrient removal (BNR). With development of the hybrid membrane aerated biofilm reactor (MABR) technology, implementation of a fully anoxic suspended growth community in BNR facilities became practical. To better understand potential limitations with the elimination of aeration, we carried out microscopic examination and 16S rRNA gene‐based microbial community profiling to determine how an anoxic suspended growth would differ from the conventional aerobic process in floc characteristics, microbial diversity, microbial temporal dynamics, and community assembly pattern. Fewer filamentous populations were found in the anoxic mixed liquor, suggesting easily sheared flocs. The anoxic microbial community had distinct composition and structure, but its diversity and temporal dynamics were similar to the conventional aerobic community. A variety of well‐studied functional guilds were also identified in the anoxic community. The anoxic microbial community assembly was more stochastic than the conventional aerobic community, but deterministic assembly was still significant with a large core microbiome adapted to the anoxic condition.

**Practitioner Points:**

- Flocs developed under the anoxic conditions had less filamentous backbones, implying reduced flocculation capacity and easily sheared flocs.
- Knowledge about the ecophysiology of *Thauera, Thiothrix,* and *Trichococcus* can help achieve good properties of the anoxic flocs.
- A diverse microbial community sustainably adapted to the fully anoxic condition, containing a variety of filaments, denitrifiers, and PAOs.
- The anoxic microbial community displayed a similar degree of diversity and temporal dynamics compared to the aerobic counterpart.
- The anoxic community's assembly was more stochastic, so it may be less subject to changes in environmental variables.

## Graphical abstract

This paper investigated that how an anoxic suspended growth process would differ from a conventional aerobic counterpart in floc characteristics, microbial diversity, temporal dynamics, and community assembly processes. We also revealed potential limitations with the elimination of aeration in the suspended growth for the biological nutrient removal processes.

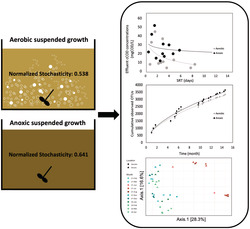

## INTRODUCTION

Anoxic conditions (zones) are routinely incorporated in suspended growth processes for biological nutrient removal (BNR). Nitrogen removal occurs through the denitrification pathway in the anoxic zones of these processes by a mix of heterotrophic bacteria that metabolize organic carbon using nitrate, nitrite, and intermediates as electron acceptors. Fully anoxic suspended growth systems have been used to degrade xenobiotics in industrial wastewater (Bajaj et al., 2010; Moussavi et al., 2014) and nitrate‐enriched marine recirculating aquaculture wastewater (Y. Gao et al., 2020; Letelier‐Gordo & Martin Herreros, 2019). These results suggested that heterotrophic denitrification can be an economically and environmentally sustainable wastewater treatment strategy compared to aerobic carbon oxidation, as it produces less biomass to be handled and eliminates aeration energy.

Although separate stage anoxic suspended growth processes exist treating a nitrified secondary effluent, fully anoxic suspended growth systems directly treating municipal wastewater are very rare. Essentially, all conventional BNR facilities incorporate aerobic zones for nitrification. Due to the slow growth rates of nitrifying bacteria, the aerobic zone usually accounts for the largest portion of the bioreactor volume in municipal wastewater treatment plants. Maintaining a large aerobic zone is not desirable, however, because it increases the extent to which influent carbon is oxidized. In fact, adding a supplemental carbon source is a common practice to maintain sufficient denitrifying activity and meet low effluent total nitrogen (TN) limits. Elimination of the aerobic zone in a suspended growth BNR process can significantly reduce the influent carbon required to meet a specified effluent TN limit, thereby reducing or eliminating the need for supplemental carbon and allowing an increased proportion of the carbon entering the wastewater treatment plant to be captured for other purposes, such as energy production (Carlson et al., 2021; Daigger et al., 2019). More complex BNR strategies, such as biological phosphorus removal, could also benefit from aeration reduction. The activities of denitrifying polyphosphate‐accumulating organisms (PAOs) under anoxic conditions have also been documented (Carvalho et al., 2007, Díez‐Montero et al., 2016, Filipe & Daigger, 1999, Gao et al., 2017), indicating that biological phosphorus removal can also be achieved in a fully anoxic suspended growth process.

The development of hybrid membrane aerated biofilm reactor (MABR) technology in recent years provides a practical alternative to an aerated suspended growth bioreactor for nitrification. It is well demonstrated that incorporation of an appropriately sized MABR in the unaerated suspended growth zone of activated sludge can provide additional surface area for a nitrifying biofilm to produce nitrate for subsequent denitrification in the bulk mixed liquor (He et al., 2021; Lu et al., 2020). In a computer model simulation of the hybrid BNR/MABR process treating wastewater characteristic of domestic sewage, high‐quality effluents were achieved despite 98% of the total bioreactor volume being unaerated (anaerobic zone of 12%, anoxic zone of 86%), leaving a 2% aerobic zone for final polishing (Carlson et al., 2021). Energy requirements were also reduced substantially as less carbon was needed for BNR, and the oxygen needed for nitrification was transferred much more efficiently with MABR than with conventional suspended growth oxygen transfer systems.

While modeling exercises can provide proof of concept that anoxic suspended growth processes can provide significant advantages, today's state‐of‐the‐art activated sludge mathematical models do not incorporate information on microbial composition, structure, and dynamics. These models describe the microbial community based on principal metabolisms (e.g., nitrification and denitrification), and “lump” the microorganisms into functional groups, namely, ordinary heterotrophs, autotrophs, PAOs, and glycogen‐accumulating organisms (GAOs) (Henze et al., 2000). Overall treatment performance is then linked to these metabolisms using mathematical equations. As biological activities of the microbial communities are the foundation for the biological transformations in suspended growth processes and directly link to the BNR performance, efforts to understand microbial composition, structure, and dynamics are critical to improving treatment processes.

Consequently, the characteristics of anoxic suspended growth biomass require further investigation to demonstrate that a viable suspended growth mixed liquor can be produced. It is unclear how the elimination of aeration would impact growth of the filamentous backbone and mixed liquor bio‐flocculation. The population of filamentous organisms is often reduced in low dissolved oxygen (DO) environments (Chudoba, 1985; Gabb et al., 1991). Although some filamentous bacteria can use nitrate as the electron acceptor (Wang et al., 2016), it is unclear if their growth in a fully anoxic process will be sustainable. With rare examples of fully anoxic suspended growth systems for municipal wastewater treatment, the knowledge gap must be addressed.

On‐going research comparing biological phosphorus removal under aerobic versus anoxic conditions provided the opportunity to better understand the process microbiology of an anoxic suspended growth system. We combined microscopic examination, molecular techniques, and ecological models to address questions on how an anoxic alternative would differ from an aerobic counterpart in floc characteristics, microbial diversity, temporal dynamics, and community assembly processes. We hypothesized that, while a distinct microbial community structure would develop under the anoxic condition, and the anoxic suspended growth will have more heterotrophic denitrifiers, the functional structures of the anoxic and aerobic systems may be similar. We also hypothesized that the anoxic community would be less diverse and less stochastic. Our results are important to understanding the biodiversity, functioning, and management of anoxic suspended growth, which may help to facilitate wider adoption of the anoxic suspended growth process.

## MATERIALS AND METHODS

### Bioreactor description and operating conditions

This study was carried out in two bench‐scale suspended growth sequencing batch reactors (SBRs) constructed at Ann Arbor Wastewater Treatment Plant (AAWWTP), USA. The working volume of each SBR was 6.5 L. The SBRs were inoculated with biomass from the full‐scale plant and fed with the primary effluent from the plant. One SBR, identified as the anoxic bioreactor, was operated in anaerobic‐anoxic cycles with external nitrate dosed in the anoxic reaction phase. DO concentrations in the anoxic bioreactor were not detected (WTW Multi 3420, WTW GmbH, Germany), and the closed mass balance of chemical oxygen demand (COD) to nitrate indicated negligible interference with oxygen in the anoxic bioreactor (data not shown). The other SBR, identified as the control aerobic bioreactor, was operated in parallel with conventional anaerobic‐aerobic cycles. Oxygen was the electron acceptor and was delivered in the aerobic reaction phase via a fine bubble air diffuser. In general, the SBR cycles included feed (5 min, 4 L of the working volume), anaerobic reaction (36.7 min), anoxic or aerobic reaction (167.8 min), waste (5 min), settle (20 min), decant (5 min, 4 L of the supernatant), and idle (0.5 min). Each SBR was provided with a stirrer (Xin Da Motor Co., LTD) with a variable speed to ensure good mixing conditions.

Monthly average SRTs of the bioreactors from December 2020 to April 2022 were illustrated in Figure S1. SRTs were varied in the same manner as Carlson et al. (2021), to establish critical operating parameters for biological phosphorus removal. During this operational period, the mixed liquor suspended solid (MLSS) concentrations were 1,434 ± 153 mg TSS/L for the aerobic bioreactor and 1,263 ± 380 mg TSS/L for the anoxic bioreactor. The bioreactor temperature was stable at 18°C.

### Floc morphology and filaments examination

Multiple mixed liquor samples from the bioreactors were collected from March to September 2021. Following proper sample handling and staining methods (Jenkins et al., 2003), observations were made using light microscopy under direct illumination (Zeiss Axioplan EL‐Einsatz, White plains, NY, USA). General floc properties (e.g., size, shape, and structure) and filament index were recorded following the protocols by Eikelboom, 2000. The circumscribing diameter was used to define the floc size (Jarvis et al., 2005), and the measurement was performed in Carl Zeiss AxioVision Rel. 4.7. Filaments that protrude from the floc were not included when establishing the circumscribing diameter. 10 flocs of each sample were randomly selected and measured to calculate the average floc size. Filamentous bacteria identification was based on both morphotypes and 16S rRNA gene sequencing data.

### DNA extraction, PCR amplification, and Illumina sequencing

A total of 42 mixed liquor samples were collected from February 2021 to April 2022 for 16S rRNA gene sequencing. All samples were stored in −80°C before sequencing. DNA was extracted with the Maxwell 16 LEV blood DNA kit (Promega Corporation, WI, USA) following the modified protocol developed by Pinto et al. (2012). The prepared extractions were submitted to the University of Michigan Microbiome Core for 16S rRNA gene sequencing. Sequencing was performed on the Illumina MiSeq platform (Illumina Inc., San Diego, CA, USA) in a 500v2 Full Flow Cell. 515F/806R primers were used to target the V4 region of 16S rRNA genes (Walters et al., 2016).

### Statistical analysis

#### Community composition, structure, and diversity analysis

Sequence processing and analysis were performed using Mothur (version 1.45.3) (Schloss et al., 2009), following the protocol outlined in Kozich et al., 2013. Sequences were aligned to the customized Silva database of V4 region, and sequences that did not align to the correct region were culled. A pseudo‐single linkage algorithm was used to further de‐noised the sequences by allowing for up to two differences between sequences. In the resulting sequences, chimeras were removed via the VSEARCH algorithm in Mothur (Rognes et al., 2016). The remaining quality sequences were classified with the MiDAS 4 reference database (Dueholm et al., 2022), with a threshold confidence level of 80% (Wang et al., 2007). Sequences with unknown domain level of taxonomy were not included, and the rest were clustered into operational taxonomic units (OTUs) with a similarity threshold of 97%.

Statistical comparison between the anoxic and aerobic communities was performed in R (version 4.0.5). Alpha diversity indices focusing on both evenness and richness, that is, Shannon and Simpson indices, were used to quantify microbial taxonomic diversity. Indices focusing solely on species richness were not used in this study due to their intrinsic estimation uncertainty (Haegeman et al., 2013). The Shapiro–Wilk test revealed that the Alpha diversity indices had a non‐normal distribution.

Beta‐diversity analyses were performed to measure the compositional dissimilarities between samples. Two‐dimensional Principle Coordinate Analysis (PCoA) plots based on the Bray‐Curtis metric was created to visualize the compositional dissimilarity between communities in the two bioreactors (Schloss et al., 2009). Non‐parametric analysis of molecular variance (AMOVA) was employed to determine whether the clustering within the ordinations was statistically significant (*p* < 0.05 was determined to be significant a priori) (Schloss, 2008).

#### Community temporal dynamics analysis

The taxa‐time relationship (TTR) and core microbiome were explored to assess microbial temporal dynamics in the overall bacterial assemblage in the aerobic and anoxic suspended growth communities. TTR describes the accumulation of new taxa over time via a power law model (S = cT^w^) and was used to assess species turnover in the microbial community. In the TTR model, S is the cumulative observed taxa, c is a constant, T is the time of observation, and w is the temporal scaling exponent which measures the relative species turnover rate (Guo et al., 2019; Meerburg et al., 2016; Wells et al., 2011). The core microbiome was determined based on the relative abundance and occurrence frequency of OTUs (Saunders et al., 2016). Three frequency thresholds were used for core members with >0.1% relative abundance in 80% (strict core), 50% (general core), and 20% (loose core) of all samples from each bioreactor (Dueholm et al., 2022).

#### Community temporal assembly analysis

Two types of null model analysis were employed to disentangle the stochastic and deterministic assembly. The first one was based on the method proposed by Ning et al. (2019). Normalized stochastic ratios (NSTs) and standardized effect sizes (SESs) based on the Bray–Curtis dissimilarity metric were calculated for both the anoxic and aerobic communities. NST reflects the contribution of stochastic processes to the community assembly relative to deterministic processes, based on magnitude rather than significance, and SES measures the significance of deterministic factors on community assembly. In the second null model analysis, a modified Raup‐Crick (RC) metric was calculated using the pipeline proposed by Chase et al. (2011). Non‐metric Multi‐dimensional Scaling (NDMA) plots based on the modified RC metric were created to visualize the dissimilarity between samples.

### Wet chemistry analysis

Colloidal COD (cCOD) was measured as the difference between COD of the filtrates from 1.2 and 0.45 μm filters. The measurement steps followed Method 5220 A and C of Standard Methods for the Examination of Water and Wastewater, 22nd Edition (2012) (APHA, 2012). Phosphorus measurements used the ascorbic acid colorimetric method derived from Method 4500‐P‐E of Standard Methods for the Examination of Water and Wastewater, 21nd Edition (2005) (APHA, 2005)

## RESULTS AND DISCUSSION

### Floc morphology and potential link to cCOD removal

**FIGURE 1:**
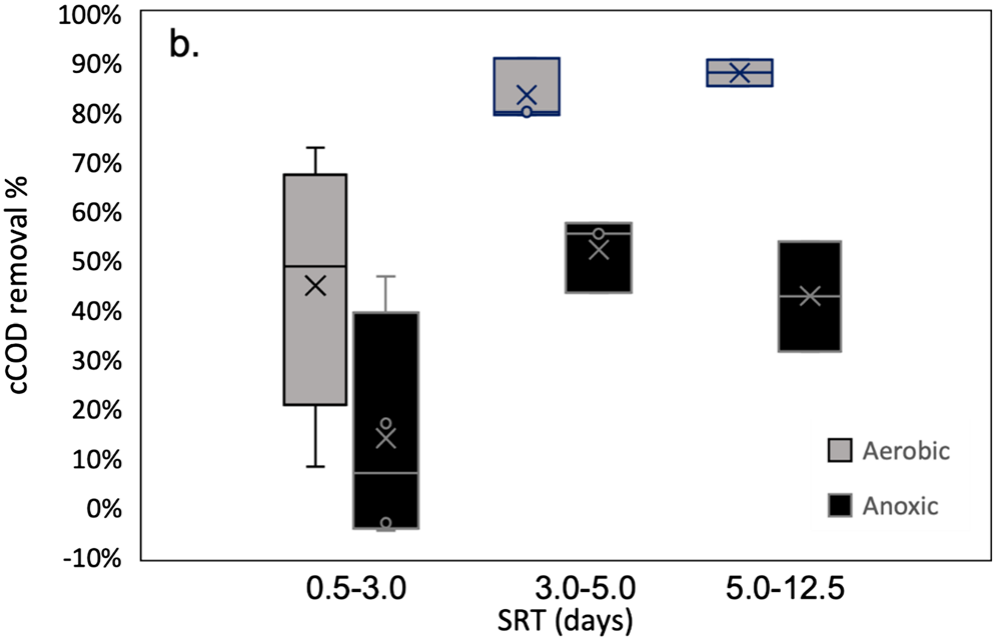
Comparison of the colloidal COD percentage removal between the aerobic and anoxic bioreactors at different SRT ranges

Microscopic examinations showed that the aerobic flocs were larger in size, stronger and more compact in structure, while anoxic flocs were smaller and less firm with internal voids and had less filaments (Figure S2). The discrepancy was consistent throughout the operational period at different SRTs (Table 1). The weaker strength was likely associated with anoxic flocs that were more susceptible to shear force, resulting in increased dispersed cells in the mixed liquor (Figure S3). Compared to the aerobic bioreactor, the anoxic bioreactor consistently had elevated concentrations of cCOD in the effluents (Figure 1a). cCOD generally represents non‐settleable particulate matter such as the dispersed solids noted microscopically. Those dispersed solids also include single cells. Furthermore, incomplete flocculation of cCOD contained in the influent wastewater is often observed for systems operating at lower SRT but is more complete as the SRT increases (Grady et al., 2011; Jimenez et al., 2005), a trend that is observed for both bioreactors (Figure 1b).

**TABLE 1:** Floc sizes and filamentous index of mixed liquor samples at different SRTs based on the microscopy examination

Sample type	SRT (days)	Average floc size (μm)	Filamentous index
Aerobic	3.6	240.7 ± 40.9	2.5
	4.6	241.1 ± 97.4	2.5
	10.1	265.3 ± 112.2	2
Anoxic	3.2	155.4 ± 42.7	1.5
	4.7	166.2 ± 93.5	1.5
	11.2	185.8 ± 79.6	1

### Filamentous bacteria identification

**FIGURE 2:**
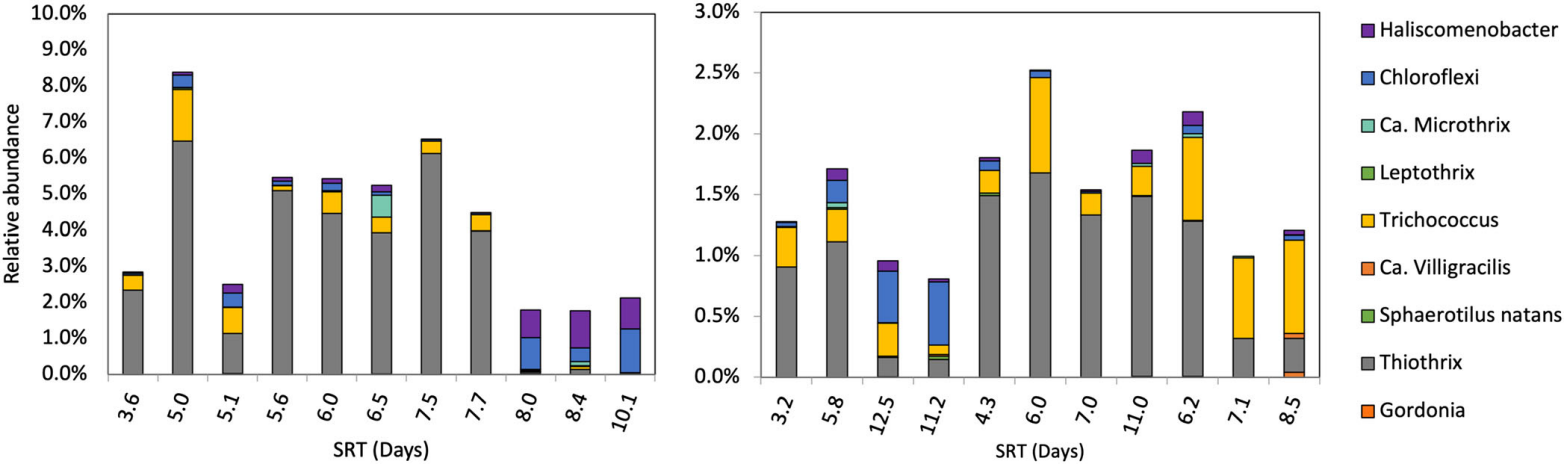
Relative abundances of known filamentous organisms in aerobic and anoxic suspended growth communities at different SRTs

Filamentous bacteria are essential backbones to form strong flocs needed for optimal performance. In this study, over 20 genera known to contain filamentous species were screened in the 16S rRNA gene sequencing data for both bioreactors at different SRTs (Figure 2). Consistent with microscopic observations, the anoxic suspended growth contained fewer filaments than the aerobic one throughout the operational period. Mean relative abundances with standard deviation (SD) of the identified filaments were 4.23 ± 2.19% in the aerobic community and 1.53 ± 0.54% in the anoxic community.

*Thiothrix* were predominant in both aerobic and anoxic bioreactors. *Thiothrix* are mixotrophs and can use organic acids as well as H_2_S as electron donors (Ravin et al., 2021). The filamentous *Trichococcus*, possibly corresponding to the frequently observed Nostocoida limicola morphotype (Nielsen et al., 2009), were also abundant in anoxic samples. Previous study reported that *Trichococcus* were very common in the influent wastewater (Dottorini et al., 2021), which may have contributed to their high abundances in the anoxic bioreactor. *Haliscomenobacter* filament type was abundant in the aerobic samples (mean ± SD = 0.31 ± 0.38%) but less represented in the anoxic samples (0.05 ± 0.04%). *Haliscomenobacter* are strictly aerobic heterotrophs and their low abundance in the anoxic samples might be attributed to the immigration via the source community in the influent wastewater (Gonzalez‐Martinez et al., 2016; van Veen et al., 1973).

Successful operation of AS systems requires a balanced composition of filamentous bacteria to ensure a good bio‐flocculation and strong flocs. With the well‐studied growth kinetics and habitats of filamentous bacteria (Chudoba, 1985; Jenkins et al., 2003; Nittami & Batinovic, 2022), identifying which filamentous bacteria can sustainably grow under anoxic conditions helps develop control strategies for anoxic suspended growth applications. For example, the Nostocoida filament type, which may translate to *Trichococcus* in this study (Nielsen et al., 2009), favors moderate to long SRTs; therefore, if excessive growth occurred, one approaching for controlling its growth is to reduce the SRT (Grady et al., 2011). However, unlike conventional AS processes where bulking and foaming are major operational problems (Eikelboom, 2000; Jenkins et al., 2003; Nittami & Batinovic, 2022), the anoxic suspended growth may face the issue of insufficient filaments and thereby easily sheared flocs. One standard approach applied in BNR systems when insufficient filaments are present is to provide a modest level of aeration in the anoxic zone to encourage the controlled growth of a modest fraction of low‐DO filaments (Jenkins et al., 2003). Such a strategy could be pursued in a largely anoxic system.

### Microbial community structure and recurring seasonal pattern

**FIGURE 3:**
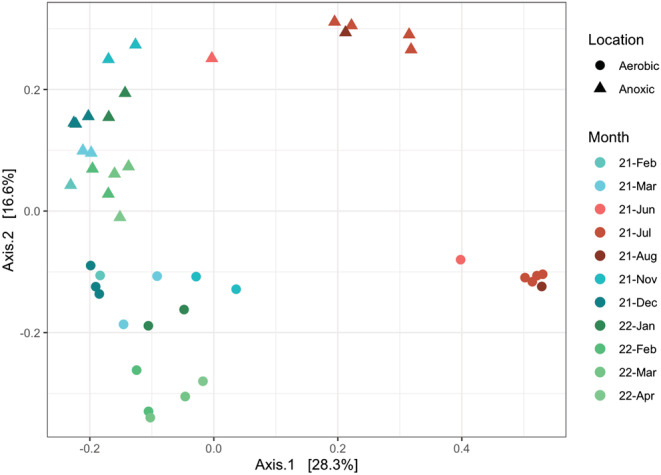
PCoA plots for the anoxic and aerobic suspended growth

PCoA analysis showed a clear clustering of aerobic and anoxic bioreactor samples (Figure 3), suggesting that the anoxic and aerobic suspended growth developed distinct microbial structures (AMOVA test *p* < 0.001). Both anoxic and aerobic suspended growth communities exhibited clear seasonal cycling and potential annual reproducibility in the microbial structure. Specifically, samples collected in seasons from the first year clustered with the samples from corresponding seasons of the following year. In addition, compared to the clusters of samples collected in cold seasons, the clusters of warm season samples from one bioreactor were more distant from the clusters of the other bioreactor, indicating that the two communities became more distinct in the warm season. Seasonal patterns have been observed in many full‐scale WWTPs, and process tank temperature was generally believed to be an important factor driving community dynamics (Flowers et al., 2013; Peces et al., 2022; Roy et al., 2017). Process tank temperature was unlikely a key factor in this study, however, as both bioreactors were operated at a constant temperature year‐round. Considering the suspended growth community and influent wastewater community as a unique entity, we hypothesize that the yearly variation and seasonal occurring pattern observed in the bioreactors were due to seasonal variations in wastewater characteristics and immigration via the influent microbial community, which was indeed affected by seasonal temperatures. It was likely that certain obligate aerobes and anaerobes had a narrow temperature range for their optimal growth, and their abundances in the influent peaked during warm seasons. After they were transported into the bioreactors, they thrived as a result of species sorting carried out by aerobic or anoxic operations, thereby resulting in different compositions and structure between the aerobic and anoxic communities. The observed ordination pattern may also result from alternating wet and dry seasons during the cold and warm months (Wágner et al., 2022). Seasonal rainfall events may change the properties of the influent wastewater which affected the microbial compositions. A detailed investigation of seasonality was not performed in this study, and future research is in need to explore the linkages between environmental variables, influent communities, and microbial seasonal dynamic patterns.

Overall, the two bioreactors used the same inoculum and received the same feed wastewater, but their microbial communities developed distinct structures. This may not be surprising, considering the apparent differences in aerobic and anaerobic metabolisms, but a detailed analysis regarding their microbial compositions (discussed in later sections) provides insight into what functional organisms appear under different conditions leading to performance optimization. Even though random processes of birth/death, speciation/extinction, and immigration can also influence community structure and lead to measurable differences (Dottorini et al., 2021; Ofiţeru et al., 2010; Zhou et al., 2013), random factors alone cannot explain the ordination pattern observed in this study. Differences between the aerobic and anoxic microbial communities were consistent over time, and their responses in different seasons were consistently distinct.

### Microbial composition and taxonomic diversity

A total of 3,320 and 3,582 taxa in the Bacteria were found in the aerobic and anoxic communities. Subsampling at a library size of 7,537 sequences captured the majority of the richness for samples, with coverage estimation of 95.9  ± 0.5%. The two communities showed a similar level of Alpha diversity (Shannon *p* = 0.45 and Simpson *p* = 0.36) (Table 2). This similarity indicates that other selective forces besides electron acceptors, such as anaerobic and aerobic/anoxic cycling, plug flow‐like conditions in the bioreactor, and SRTs, impacted species diversity.

**TABLE 2:** Diversity indices of microbial compositions in bioreactors

Bioreactor type	Shannon	Simpson
Aerobic	5.03 ± 0.33	0.98 ± 0.02
Anoxic	5.02 ± 0.18	0.98 ± 0.01

Both aerobic and anoxic communities were dominated by OTUs affiliated with Proteobacteria (36.80 ± 4.81% for the aerobic community; 36.09 ± 4.58% for the anoxic community), followed by Bacteroidota (28.77 ± 6.44%; 29.55 ± 3.68%) and Campylobacterota (8.84 ± 6.47%; 13.14 ± 7.39%). Proteobacteria and Bacteroidota are common predominant phyla present in municipal wastewater treatment plants (WWTPs) (Dueholm et al., 2022; Wu et al., 2019). They play a key role in nitrogen cycling and organics degradation (Nascimento et al., 2018). Other common phyla reported elsewhere, such as Actinobacteriota and Chloroflexi, showed low relative abundances in this study. Campylobacterota, which is less reported in AS studies, were abundant in both communities, especially during the cold season from November 2021 to March 2022 (11.93 ± 4.89%; 16.71 ± 5.14%). Most of them belong to the family *Arcobacteraceae*, which can be seen as a waterborne pathogen and can cause human illness (Venâncio et al., 2022). Reasons behind their high relative abundance during the cold seasons are not clear, but previous studies suggested their presence correlates with high levels of fecal contamination (Collado et al., 2008; Lee et al., 2012).

The top abundant OTUs (mean relative abundance across the time‐series samples >1%) were shown in Figure S4. Comparison indicates that some species were with strong preference for either aerobic or anoxic conditions. The high abundance of OTU0013 (Genus: *Zoogloea*) (1.59 ± 1.66%) in the aerobic community agreed with the larger size of flocs developed in the aerobic reactor. *Zoogloea* can use both oxygen and nitrate as electron acceptors but grows faster in aerobic environments, so their proliferation in the aerobic bioreactor was logical. OTU0025, identified as the genera *Lentimicrobium*, was only abundant in the anoxic community. *Lentimicrobium* is an anaerobic bacterium, so they selectively proliferated in the oxygen‐free conditions. Besides obligate organisms that were exclusive in one community over another, the anoxic and aerobic communities also shared common abundant species, that is, OTU0001 (Genus: *Arcobacter*), OTU0002 (Genus: *Pseudarcobacter*), OTU0004 (Family: *Comamonadaceae*), OTU0003 (Genus: *Pseudarcobacter*), OTU0008 (Family: *Hydrogenophilaceae*). Those species, if growing, are likely to be process‐critical species that have facultative heterotrophic denitrification capability. It is also possible that some shared abundant species were simply due to mass immigration with the influent source community (Dottorini et al., 2021). Continuous transport into the bioreactors from upstream influents, that is, plant primary effluent, may have kept them abundant despite inactivity during the actual treatment stage. *Arcobacter*, for example, was reported among the incoming highly abundant genera in WWTPs but non‐growing in the process tank (Kristensen et al., 2020).

### Microbial community temporal dynamics

**FIGURE 4:**
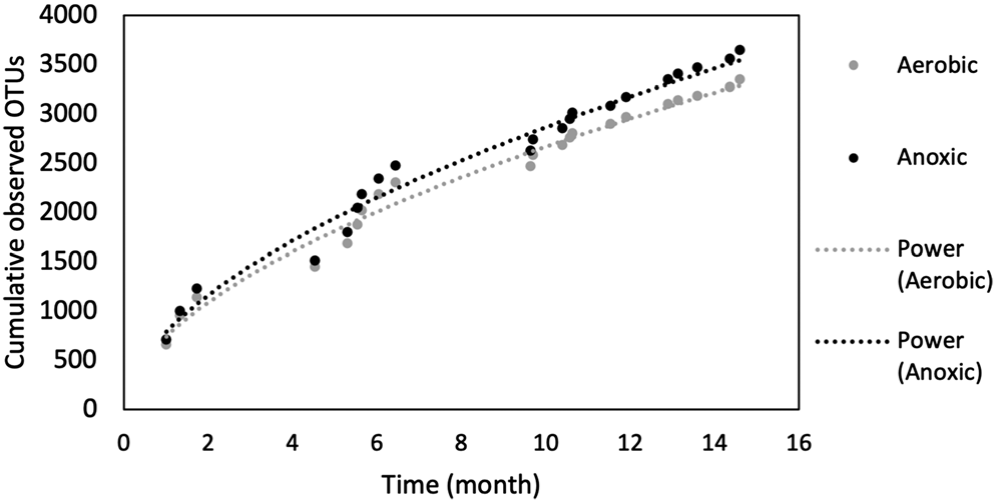
Taxa‐time relationship and least‐squares nonlinear regression power law fit between time and cumulative observed OTUs in the aerobic and anoxic bioreactors

The bacterial TTR of the two communities was examined using the power law equation (S = cT^w^) to characterize how the species changed with time. Cumulative observed OTUs were fitted to the equation to determine the steepness of the TTR slope (w), which measures the relative species turnover rate. In this study, the power law model displayed a strong fit (*R* ^2^ > 0.97) to the molecular characterization of community dynamics (Figure 4). The calculated exponent values were 0.561 for the anoxic and 0.556 for the aerobic community. Both values fell within the typical ranges reported previously (Guo et al., 2019; Hai et al., 2014; van der Gast et al., 2008).

TTR models have been used as informative indicators of the ability of a microbial community to support and maintain a balanced assemblage of organisms, including decreasing temporal scaling exponents responding to increasing selective pressure (Rivett et al., 2021). Species turnover rates can be influenced by community diversity and the spatial scale of observation. Theory predicts that the decline in species turnover occurs in more diverse communities that have greater temporal stability, as was validated in previous microbial studies (Hai et al., 2014; Rivett et al., 2021). Overall, the exponent values for the aerobic and anoxic communities were remarkably close (*p* > 0.05), suggesting a similar species turnover along the time span over which the communities were observed.

The core microbiome also reflects microbial temporal dynamics as it describes a community that is constantly associated with a given environment (Neu et al., 2021; Xia et al., 2018). In this study, we identified core members in both the anoxic and aerobic suspended growth communities. The anoxic suspended growth community contained 218 loose core taxa (79.71 ± 5.61%), 98 general core taxa (60.34 ± 15.47%), and 41 strict core taxa (41.50 ± 12.17%). In the aerobic community, there were 247 loose core genera (78.81 ± 8.00%), 103 general core genera (56.59 ± 16.81%), and 28 strict core genera (28.20 ± 6.62%). Results showed that a large persistent population adapted to the anoxic condition imposed. The presence of core microbiome is important in modeling to predict and diagnose process functions for anoxic suspended growth, as persistent populations (especially persistently abundant organisms) may make essential contributions to treatment performance and temporal stability (Saunders et al., 2016).

### Diversity within functional guilds

**FIGURE 5:**
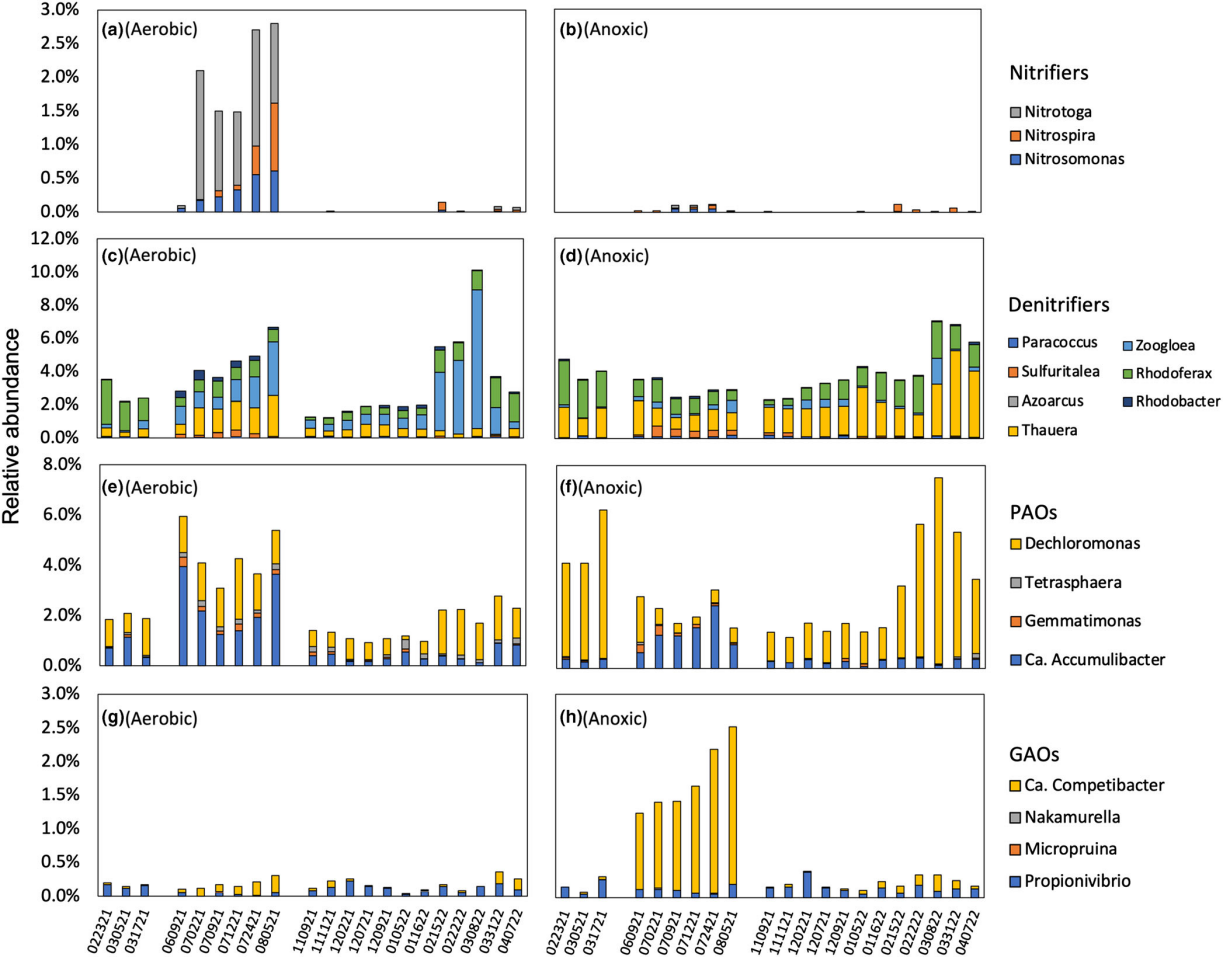
Diversity of genera belonging to major functional groups. The *x* axis indicated the sampling dates (e.g., 022321 = February 23, 2021).

Insight into which bacteria are responsible for nutrient removal is critical to improve BNR processes, particularly anoxic BNR processes as there is such little information about them in full systems. Therefore, the taxonomy diversity of well‐described nitrifiers, denitrifiers, PAOs, and GAOs was examined in this study. As the bioreactors were not designed to nitrify, the relative abundances of nitrifiers were less than 0.04% in most of the samples (Figure 5a & 5b). An enrichment of nitrifiers occurred in the aerobic community during the summer months (2.12 ± 0.63%) with both abundant AOBs (*Nitrosomonas* and *Nitrospira*) and NOBs (*Nitrospira* and *Nitrotoga*). This can be attributed to the fact that the growth rate of nitrifiers peaked during the summertime, and the SRT of the aerobic bioreactor (8–10 days) during that period was much longer than typical washout conditions. It is well‐known that nitrification is an “all or nothing” proposition (Grady et al., 2011), and nitrate production may have interfered with the preceding anaerobic phase in the aerobic bioreactor at that time (data not shown). However, the nitrifiers were washed out after the SRT was reduced. The presence of low abundant nitrifiers in the anoxic bioreactor was likely due to immigration via the influent wastewater. Nitrate concentrations were consistently low enough (<1 mg‐N/L) during the anaerobic phase in the anoxic bioreactor and were not considered to have interfered with anaerobic functions.

Among the known genera, denitrifiers made up of 3.25 ± 2.31% and 3.48 ± 1.68% for the aerobic and anoxic communities (Figure 5c,d). *Zoogloea* (1.59 ± 1.66%) and *Rhodoferax* (0.96 ± 0.62%) were the top abundant genera in the aerobic bioreactor, while *Thauera* (1.90 ± 1.07%) and *Rhodoferax* (1.30 ± 0.67%) were most abundant in the anoxic bioreactor. Besides being important to the denitrification process, both *Zoogloea* and *Thauera* are also known as floc‐formers (Thomsen et al., 2007). Compared to *Zoogloea*, *Thauera* are more versatile in use of available substrates such as sulfur species and are, thereby, more capable of living in an oxygen‐free environment. A previous study reported that *Thauera* can utilize sulfide as the electron donor for denitrification and store intracellular elemental sulfur (Liang et al., 2020). Sulfur granules were also observed in the anoxic biomass samples by microscopic examination. Deeper knowledge about the ecophysiology of *Thauera* may ensure the presence of denitrification and help control for settleable floc properties in anoxic suspended growth.

Biological phosphorus removal is accomplished by PAOs, with four genera identified in this study (Figure 5e,f). Ca. Accumulibacter and *Tetrasphaera*, two well‐described PAO genera that have received intensive investigations (S. He et al., 2007; Marques et al., 2017; Nguyen et al., 2011; Stokholm‐Bjerregaard et al., 2017), were less represented in the anoxic bioreactor. The relative abundance of Ca. Accumulibacter in the anoxic bioreactor was 0.52 ± 0.53% while the number in the aerobic bioreactor was almost double (1.02 ± 1.09%). *Tetrasphaera* accounted for only 0.03 ± 0.04% of the total population in the anoxic bioreactor but five times more (0.15 ± 0.09%) in the aerobic bioreactor. The denitrification capability of different Ca. Accumulibacter clades have been well‐documented (Carvalho et al., 2007; Díez‐Montero et al., 2016; Filipe & Daigger, 1999; Flowers et al., 2009; H. Gao et al., 2019). While most previous studies used synthetic wastewater, Díez‐Montero et al., 2016 recorded the evolution of denitrifying PAOs in an anaerobic‐anoxic sludge blanket treating municipal wastewater. Our study revealed the feasibility to enrich denitrifying PAOs in the suspended growth for municipal wastewater treatment. Process optimization and finer‐resolution‐diversity of the denitrifying PAOs will need future investigations.

The recognized putative PAO genera *Dechloromonas* were abundant with high occurrence frequencies in both bioreactors. While members of *Dechloromonas* have been shown to behave according to the PAO phenotype, a potential GAO phenotype in situ was also reported previously (McIlroy et al., 2016; Nielsen et al., 2019). Metabolic information retrieved from metagenome‐assembled genomes revealed glycogen accumulation genes encoded in *Dechloromonas* members, suggesting the possibility that *Dechloromonas can* exhibit different metabolisms (polyphosphate‐ and glycogen‐based phenotypes) depending on environmental conditions (Petriglieri et al., 2021). A high abundance of *Dechloromonas* was identified in the anoxic bioreactor, and future analysis needs to investigate their metabolism under the anoxic condition.

Low relative abundance of recognized GAOs was found in both bioreactors (Figure 5g,h), suggesting that GAOs were not as competitive as PAOs. Although the relative abundance of Ca. Competibacter in the anoxic bioreactor surged during the summer of 2021, a moderate phosphorus percentage removal of 58.0% was still achieved in the anoxic bioreactor during that period.

### Stochastic and deterministic processes controlling microbial temporal dynamics

**FIGURE 6:**
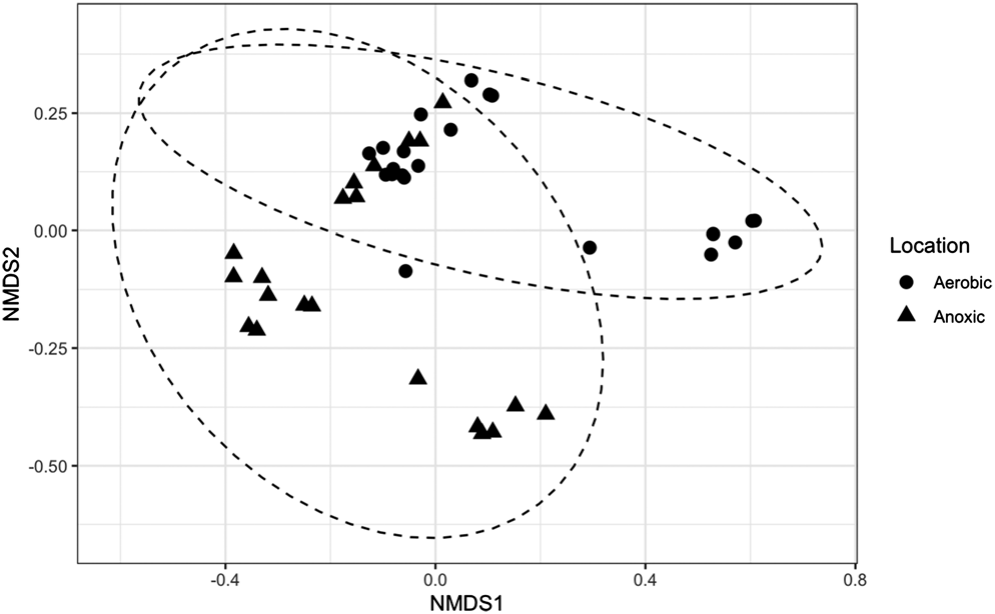
NMDS plots based on the modified RC metrics. Stress: 0.16

Microbial temporal dynamics is shaped by both stochastic (e.g., random birth/death, immigration) and deterministic (e.g., niche‐related selection) processes (van der Gast et al., 2008; Zhang et al., 2022; Zhou & Ning, 2017). Understanding the mechanisms (stochastic vs. deterministic) governing community diversity and dynamics is important to manage and control the microbial community. In the context of environmental engineering of practical biological treatment systems, determining which mechanisms are driving factors is desired to help better optimize system performance. To disentangle the stochastic and deterministic assembly in this study, two types of null model analysis were employed. In the first null model analysis, NST and SES values were calculated (Table 3). The value of NST for the anoxic community (64.1%) was higher than that for the aerobic community (53.8%), suggesting higher stochasticity in assembly of the anoxic community. A similar result was revealed by the modified RC metrics based NMDS plots (Figure 6). Samples from the anoxic bioreactor were clustered farther apart, indicating that they were less deviant to the null expectation and more shaped by stochastic factors (Zhou et al., 2014).

**TABLE 3:** Normalized stochasticity (NST) and standard effect size (SES) values for the aerobic and anoxic suspended growth

Group	Normalized stochasticity (NST)	Standard effect size (SES)
Aerobic	0.538	3.59
Anoxic	0.641	2.79

One might expect that microbial temporal assembly in anoxic suspended growth would be more niche‐related and less random than in the aerobic community, as the presence of nitrate as the electron acceptor may create a higher selective pressure, filtering out microorganisms incapable of anaerobic metabolisms. However, the null model analysis indicated that the anoxic community assembly was more stochastic than aerobic community assembly. This could be attributed to the occasionally longer SRTs in the anoxic bioreactor. It has been reported that stochastic processes generally become more pronounced at long SRTs, possibly due to the increased microbial immigration rates from the feed wastewater (Zhang et al., 2022). Long SRTs could intensify nutrient competition between functional groups and weaken their ability to resist external disturbances (Sun et al., 2020). Thus, the anoxic community occasionally operated at longer SRTs could be more disturbed by the continuous immigration from the influent wastewater. In addition, lower net specific growth rates in the anoxic bioreactor may have intensified drift or fluctuations in population size due to random birth or death events (Evans et al., 2017).

On the other hand, both communities exhibited SES values of over 2 and, therefore, deterministic factors also played significant roles in regulating microbial community structures (Ning et al., 2019). The significance of deterministic assembly was also reflected by the large number of species in the core microbiome observed in both communities. However, the role of stochastic processes in anoxic microbial assembly cannot be overlooked. Compared to conventional aerobic suspended growth, random processes, such as immigration via the influent source community, were more significant in driving community assembly in the anoxic suspended growth. Therefore, the anoxic suspended growth may be less controllable than the conventional aerobic suspended growth, but it may be less subject to changes in environmental variables (pH, temperature, etc.) at the same time.

## CONCLUSIONS AND FUTURE RESEARCH NEEDS

A fully anoxic suspended growth is appealing in BNR processes due to considerable aeration reduction and improved carbon processing efficiency. With the development of hybrid MABR technology, implementation of a fully anoxic suspended growth community in the resulting hybrid process became practical. Our study comparatively investigated the anoxic microbial community compared to a conventional aerobic community via microscopic and molecular methods. Results from this study provide a basis for understanding how an anoxic alternative differs from conventional aerobic communities and may facilitate a wider adoption of the anoxic suspended growth for improving the carbon and energy efficiency of BNR processes.

Microscopic examination showed that flocs formed under the anoxic condition had less filamentous backbones and were more diffuse with internal voids, implying reduced bio‐flocculation capacity and easily sheared flocs. Future studies need to quantify the flocculation rates and determine the proper amount of oxygen for micro‐aeration that improves floc strength, flocculation capacity, and effluent quality without sacrificing the carbon processing benefits of a fully anoxic suspended growth.

*Thauera*, *Thiothrix*, and *Trichococcus* were identified as important floc‐formers and filaments in anoxic suspended growth, so deeper knowledge about their ecophysiology can help these systems achieve good floc properties. As phylogenetic diversity exists within each filament morphotype, the identification of filamentous bacteria was with uncertainties. Fluorescence in situ hybridization (FISH) with species‐targeted probes can better identify and visualize filaments, which can be pursued in future research.

Community level analysis based on 16S rRNA gene sequencing provided insights into the microbial composition and diversity of the anoxic suspended growth. Though a distinctly different microbial community adapted to the anoxic condition, it showed a similar degree of diversity and temporal dynamics compared to the conventional aerobic community. This is important because diversity and dynamics of communities are often related to functional redundancy and process stability (Briones & Raskin, 2003). Recognizing the similarities between the anoxic and aerobic communities can lead to a better integration of existing knowledge and strategies to design and control the anoxic suspended growth process.

A variety of well‐described filaments, denitrifiers, and PAOs sustainably adapted to the anoxic condition. Since bacterial identification at the species level is a challenge for 16S rRNA gene V4 region‐based analysis, some members within the functional guilds were either unclassified or poorly described (i.e., those with only a MiDAS placeholder species name). More specific and quantitative functional traits‐based methods, such as GeoChip (Shi et al., 2019) and metagenomic sequencing (Singleton et al., 2021), are needed in the future research to identify the process‐critical and novel members on the species level.

Null model analysis in this study indicated that the anoxic community had a more stochastic assembly pattern than the aerobic community. This was likely due to the longer SRTs and lower net growth rates in the anoxic bioreactor. On the other hand, deterministic assembly was still significant in the anoxic community, and the general core microbiome encompassed 98 genera, representing a great portion of the total population. Different microbial groups may differ greatly in the assembly mechanisms, however, as some populations are under strong selection pressure while others have highly random birth/death events. This type of difference was not discerned by the whole community level analysis in this study. Future research is needed to apply individual taxa/lineages based analysis, such as phylogenetic bin‐based mull model (iCAMP) (Ning et al., 2020), to quantify the ecological drivers that govern the temporal assembly of various microbial groups.

## CONFLICT OF INTEREST

The authors declare that they have no known conflict of interest that could have appeared to influence the work reported in this paper.

## Supporting information


**Figure S1.** Monthly average SRTs of the aerobic and anoxic bioreactors from December 2020 to April 2022
**Figure S2.** Aerobic and anoxic flocs observation based on light microscopy examination (200x magnification direct illumination)
**Figure S3** Dispersed growth observed in the aerobic (a) and anoxic (b) suspended growth under 1000x magnification direct illumination. Suspended growth SRTs: aerobic = 10.1 days, anoxic = 11.2 days.
**Figure S4** The taxonomy and relative abundances of the top OTUs (mean relative abundance > 1%) in the aerobic community (a) and anoxic community (b). The x‐axis indicated the sampling dates (e.g., 022321 = February 23, 2021).

## Data Availability

Relevant data has been added to the supplementary information. Raw sequencing data and R scripts are available upon request.
