# Supplementary material for: Comparative analysis of floc characteristics and microbial communities in anoxic and aerobic suspended growth processes
Source: Water Environ Res. 2022 Dec 21;94(12):e10822. doi: 10.1002/wer.10822 (PMC10107865; doi:10.1002/wer.10822)
Supplement: Supplementary file 1 — Figure S1. Monthly average SRTs of the aerobic and anoxic bioreactors from December 2020 to April 2022 Figure S2. Aerobic and anoxic flocs observation based on light microscopy examination (200x magnification direct illumination) Figure S3 Dispersed growth observed in the aerobic (a) and anoxic (b) suspended growth under 1000x magnification direct illumination. Suspended growth SRTs: aerobic = 10.1 days, anoxic = 11.2 days. Figure S4 The taxonomy and relative abundances of the top OTUs (mean relative abundance > 1%) in the aerobic community (a) and anoxic community (b). The x‐axis indicated the sampling dates (e.g., 022321 = February 23, 2021). [file WER-94-0-s001.docx]

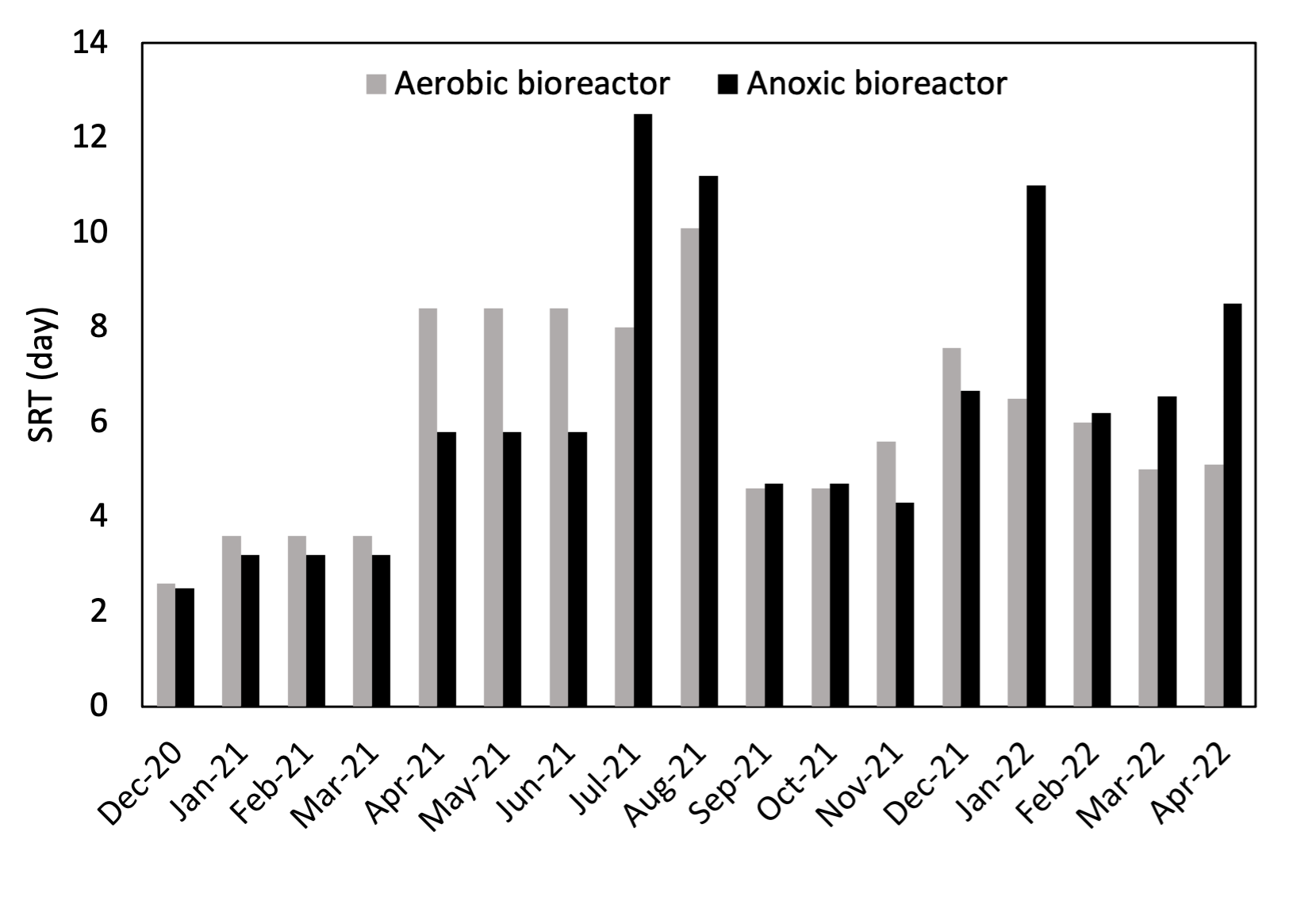


Figure S1. Monthly average SRTs of the aerobic and anoxic bioreactors from December 2020 to April 2022


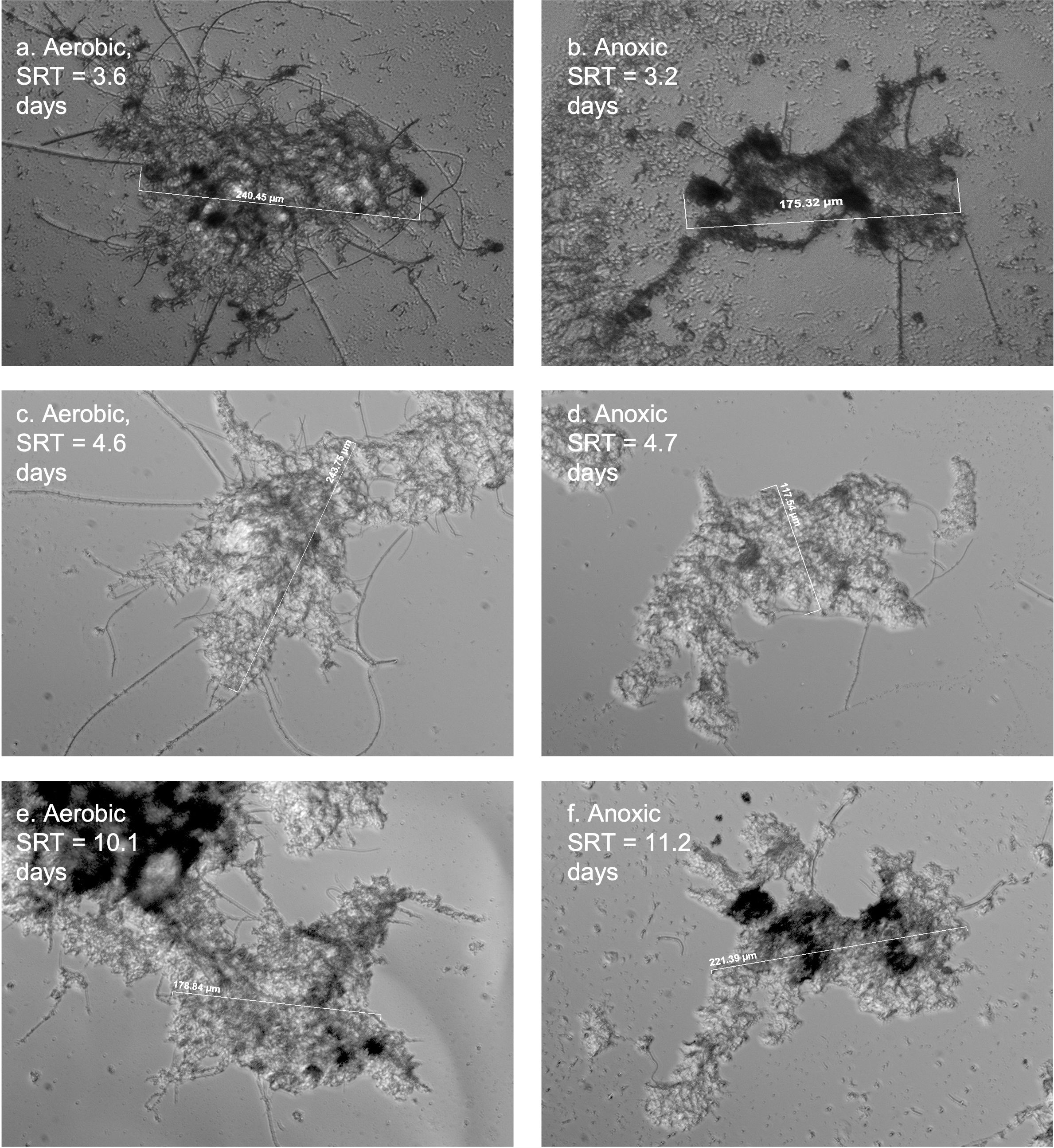


Figure S2. Aerobic and anoxic flocs observation based on light microscopy examination (200x magnification direct illumination)


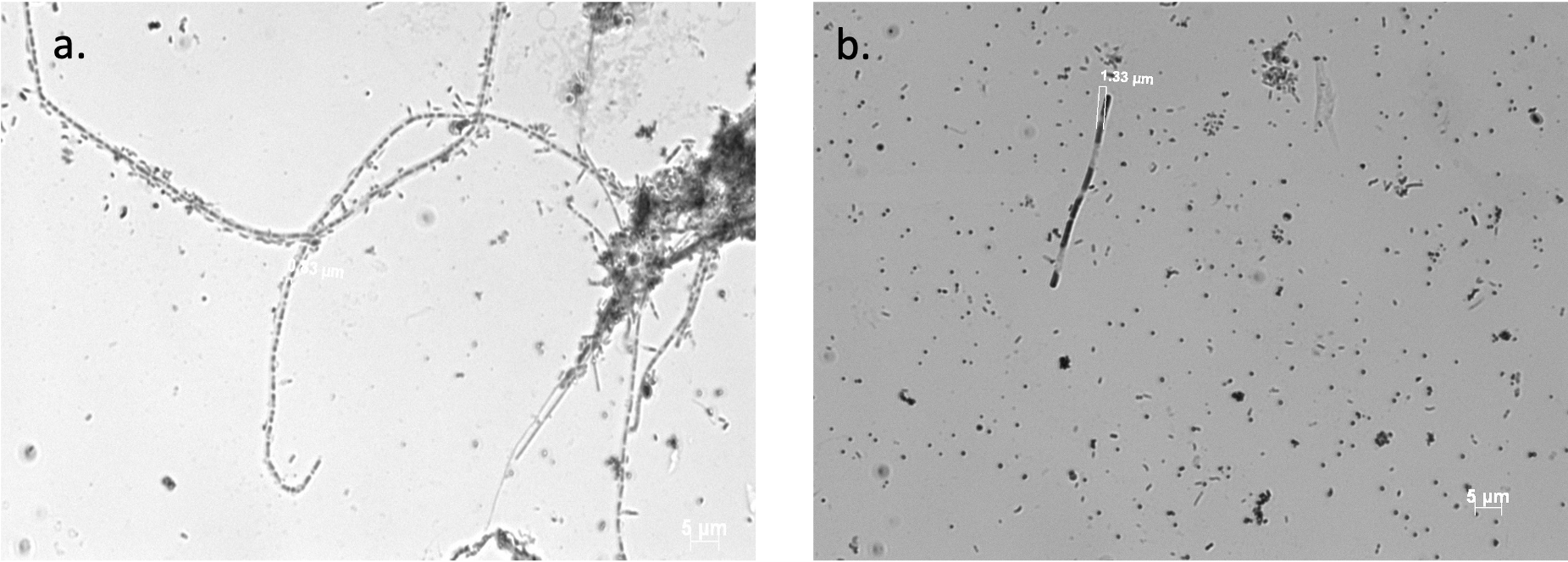


Figure S3 Dispersed growth observed in the aerobic (a) and anoxic (b) suspended growth under 1000x magnification direct illumination. Suspended growth SRTs: aerobic = 10.1 days, anoxic = 11.2 days.


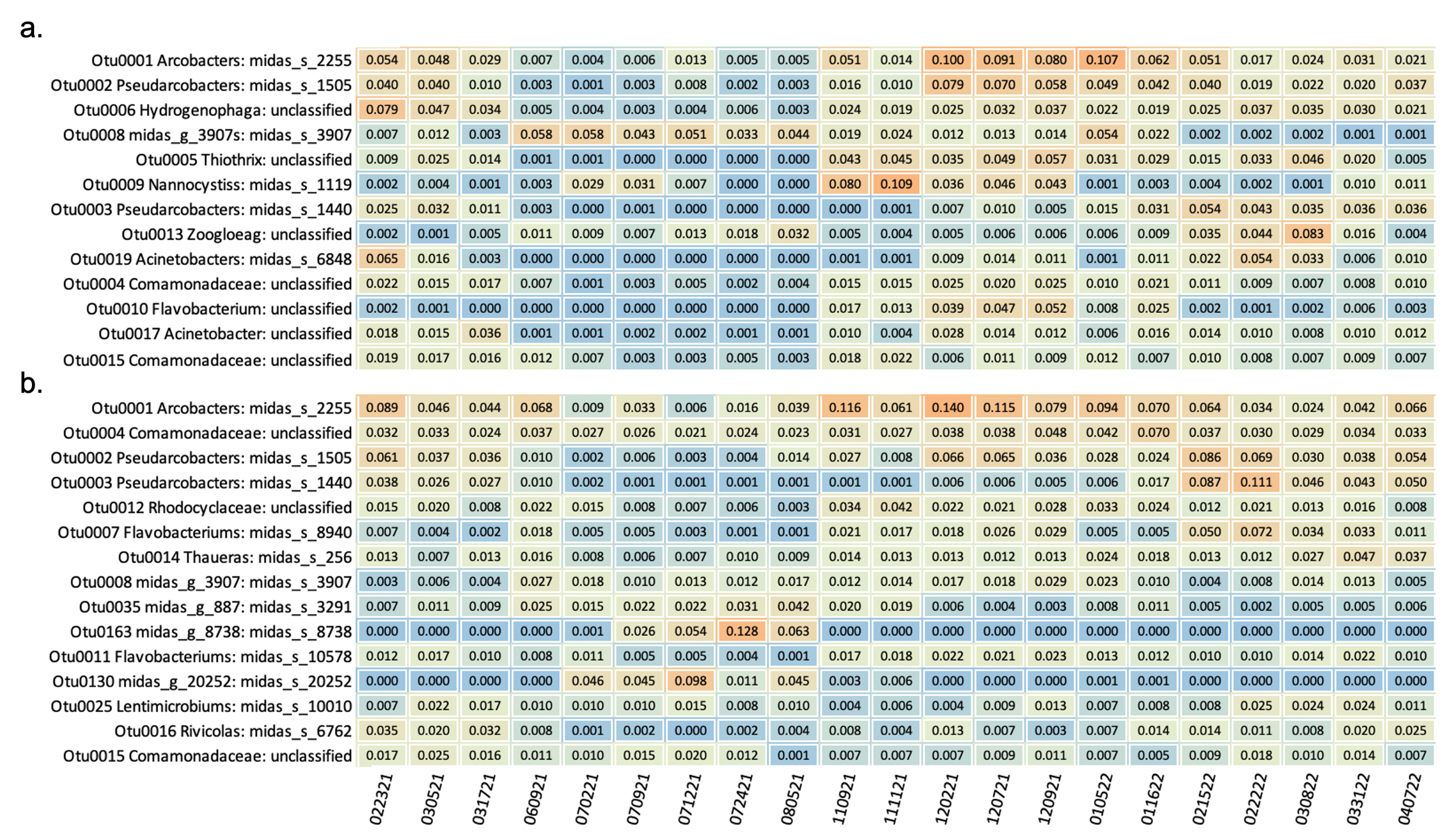


Figure S4 The taxonomy and relative abundances of the top OTUs (mean relative abundance > 1%) in the aerobic community (a) and anoxic community (b). The x-axis indicated the sampling dates (e.g., 022321 = February 23, 2021).
